# Phylogeography of mtDNA haplogroup R7 in the Indian peninsula

**DOI:** 10.1186/1471-2148-8-227

**Published:** 2008-08-04

**Authors:** Gyaneshwer Chaubey, Monika Karmin, Ene Metspalu, Mait Metspalu, Deepa Selvi-Rani, Vijay Kumar Singh, Jüri Parik, Anu Solnik, B Prathap Naidu, Ajay Kumar, Niharika Adarsh, Chandana Basu Mallick, Bhargav Trivedi, Swami Prakash, Ramesh Reddy, Parul Shukla, Sanjana Bhagat, Swati Verma, Samiksha Vasnik, Imran Khan, Anshu Barwa, Dipti Sahoo, Archana Sharma, Mamoon Rashid, Vishal Chandra, Alla G Reddy, Antonio Torroni, Robert A Foley, Kumarasamy Thangaraj, Lalji Singh, Toomas Kivisild, Richard Villems

**Affiliations:** 1 Department of Evolutionary Biology, Institute of Molecular and Cell Biology, University of Tartu and Estonian Biocentre, Tartu, Estonia; 2 Centre for Cellular and Molecular Biology, Hyderabad, India; 3 Dipartimento di Genetica e Microbiologia, Università di Pavia, Via Ferrata 1, 27100 Pavia, Italy; 4 Leverhulme Centre of Human Evolutionary Studies, The Henry Wellcome Building, University of Cambridge, Fitzwilliam Street, Cambridge, CB2 1QH, UK; 5 Students of different Universities and Colleges of India studied (as a part of their curriculum) in CCMB Hyderabad, India

## Abstract

**Background:**

Human genetic diversity observed in Indian subcontinent is second only to that of Africa. This implies an early settlement and demographic growth soon after the first 'Out-of-Africa' dispersal of anatomically modern humans in Late Pleistocene. In contrast to this perspective, linguistic diversity in India has been thought to derive from more recent population movements and episodes of contact. With the exception of Dravidian, which origin and relatedness to other language phyla is obscure, all the language families in India can be linked to language families spoken in different regions of Eurasia. Mitochondrial DNA and Y chromosome evidence has supported largely local evolution of the genetic lineages of the majority of Dravidian and Indo-European speaking populations, but there is no consensus yet on the question of whether the Munda (Austro-Asiatic) speaking populations originated in India or derive from a relatively recent migration from further East.

**Results:**

Here, we report the analysis of 35 novel complete mtDNA sequences from India which refine the structure of Indian-specific varieties of haplogroup R. Detailed analysis of haplogroup R7, coupled with a survey of ~12,000 mtDNAs from caste and tribal groups over the entire Indian subcontinent, reveals that one of its more recently derived branches (R7a1), is particularly frequent among Munda-speaking tribal groups. This branch is nested within diverse R7 lineages found among Dravidian and Indo-European speakers of India. We have inferred from this that a subset of Munda-speaking groups have acquired R7 relatively recently. Furthermore, we find that the distribution of R7a1 within the Munda-speakers is largely restricted to one of the sub-branches (Kherwari) of northern Munda languages. This evidence does not support the hypothesis that the Austro-Asiatic speakers are the primary source of the R7 variation. Statistical analyses suggest a significant correlation between genetic variation and geography, rather than between genes and languages.

**Conclusion:**

Our high-resolution phylogeographic study, involving diverse linguistic groups in India, suggests that the high frequency of mtDNA haplogroup R7 among Munda speaking populations of India can be explained best by gene flow from linguistically different populations of Indian subcontinent. The conclusion is based on the observation that among Indo-Europeans, and particularly in Dravidians, the haplogroup is, despite its lower frequency, phylogenetically more divergent, while among the Munda speakers only one sub-clade of R7, i.e. R7a1, can be observed. It is noteworthy that though R7 is autochthonous to India, and arises from the root of hg R, its distribution and phylogeography in India is not uniform. This suggests the more ancient establishment of an autochthonous matrilineal genetic structure, and that isolation in the Pleistocene, lineage loss through drift, and endogamy of prehistoric and historic groups have greatly inhibited genetic homogenization and geographical uniformity.

## Background

More than one sixth of humanity currently lives on the Indian subcontinent. This population is spread across up to 40,000 endogamous and semi-endogamous culturally, linguistically, and socially differentiated groups [1]. The majority of these groups or populations are castes, but they also include nearly 500 'scheduled tribes' [2] and ca. 500 'scheduled castes' [3]. Thus, the Indian subcontinent is an ideal region for studying the relationships between culture, geography and genes, and for developing interdisciplinary models concerning the demographic history of *Homo *sapiens or anatomically modern humans (AMH). Moreover, the large number of deep-rooting mtDNA lineages emerging from the basal nodes of both superhaplogroup M and N (including R) [4-11] indicate that the Indian subcontinent was probably the first major outcome of the dispersals of AMH from Africa. Furthermore, these deep-rooted mtDNA haplogroups generally cross cultural and social boundaries; this suggests a common origin to the highly diverse peoples of the Indian sub-continent, with indigenous or autochthonous diversification of the maternal gene pool [12-16].

These results have been generally corroborated by data from the Y chromosome [17,18] and autosomal DNA [13,19,20]. The only exception, for mtDNA, are the Tibeto-Burman speakers of north-eastern India, who share about half of their maternal genetic heritage with populations living further east of India [14,21]. It has been argued, that following the initial colonization of Indian subcontinent, maternal gene flow from the west has been rather limited and largely restricted to the western states of contemporary India and Pakistan [14,15,22]. Consequently, the haplogroup richness of the Indian subcontinent appears to have formed *in situ*, and date back to some point in the later Pleistocene, most probably between 40 Ka and 60 Ka ago. Furthermore, this high level of genetic diversity may also be linked to the possibility that the South Asian population in the Pleistocene was demographically large in global terms. Comparisons of relative regional population sizes through time, deduced by Bayesian coalescent inference methods applied to global mtDNA complete sequence data, indicate that between approximately 45 Ka and 20 Ka ago most of humanity lived in Southern Asia [23].

Two language families, Indo-European and Dravidian, account for the majority of linguistic diversity in India. However, apart from a number of linguistic isolates, there are two other major families – Tibeto-Burman and Austro-Asiatic (AA). The origin of the Austro-Asiatic language family is a highly debated issue. Building on archaeological and linguistic evidence, and the assumption that rice domestication was a single event, the currently preferred hypothesis places the origin of this language family in Southeast Asia [24-26]. The alternative model, based on genetic evidence (that shows multiple domestications of rice varieties [27]), and comparative phonology, advocates an East Indian cradle for the AA language group [28]. The AA language family tree has two basic branches – Munda and Mon-Khmer. The former is distributed exclusively in the Indian subcontinent; the latter is predominantly Southeast Asian, although there are a few Indian representatives (Khasian and Nicobarese) [26].

The genetic origin(s) of extant AA speakers, however, may or may not coincide with the origin of the language group. Studies of mtDNA diversity have shown that the AA speakers from Southeast Asia and the Indian subcontinent carry mtDNAs of different sources [7,9,14,16,29,30]. Although the data on Southeast Asian populations, which speak languages of the Mon-Khmer branch of the AA tree, are still somewhat limited, it seems safe to conclude, that their mtDNA characteristics are similar to those of the surrounding Southeast Asian populations, and distinct from AA tribes of India (Munda-speakers) [30]. Similarly, the Indian tribes speaking different Munda languages show generally the same mtDNA haplogroup composition as the Indo European and Dravidic groups of India [9,14,16,29]. In contrast, the Y chromosomes of Indian and Southeast Asian AA speaking populations share a common marker, M95, which defines a single branch (O2a) in the overwhelmingly East Asian specific tree of haplogroup O. This evidence provides a strong basis for proposing a Southeast Asian origin of the paternal lineages of the Munda speaking populations of India [13,17,18].

The AA speaking populations of Myanmar, which is a likely dispersal route, or original location, for the ancestral populations of Munda speakers of India, have not yet been sampled for their mtDNA. It is still possible that some of the mtDNA clades present among the AA speakers of India (and in their neighbours) could, in fact, be due to gene flow to India from further east. In an attempt to identify mtDNA lineages that would reveal a phylogeographic distribution similar to that of the Y chromosome marker M95, we analyzed mtDNA samples representing all the major linguistic groups of India, with a particular focus to haplogroup R derived lineages.

The first thorough study of complete mtDNA sequences from India [4] identified numerous indigenous clades emerging directly from the roots of superhaplogroups N, R and U, such as N5, R5-R8, R30, R31, U2a-d and U7. West Eurasian specific haplogroups HV, JT, N1, and U (xU2a-d, U7) occur at lower frequencies, suggesting limited but phylogeographically well detectable gene flow into the Indian subcontinent, most probably from west and northwest Eurasia [14]. Here we have now extended the complete mtDNA sequencing by determining 35 new complete sequences, in order to further refine the phylogeny of the Indian subcontinent-specific segment of haplogroup R. Furthermore, to explore the correlations between genes, languages and geography in Indian subcontinent, we have carried out high resolution genotyping and phylogeographic detailed analyses on R7, which occurs at high frequency among the Austro-Asiatic (Munda) speaking groups of India.

## Results and Discussion

**Figure 1:**
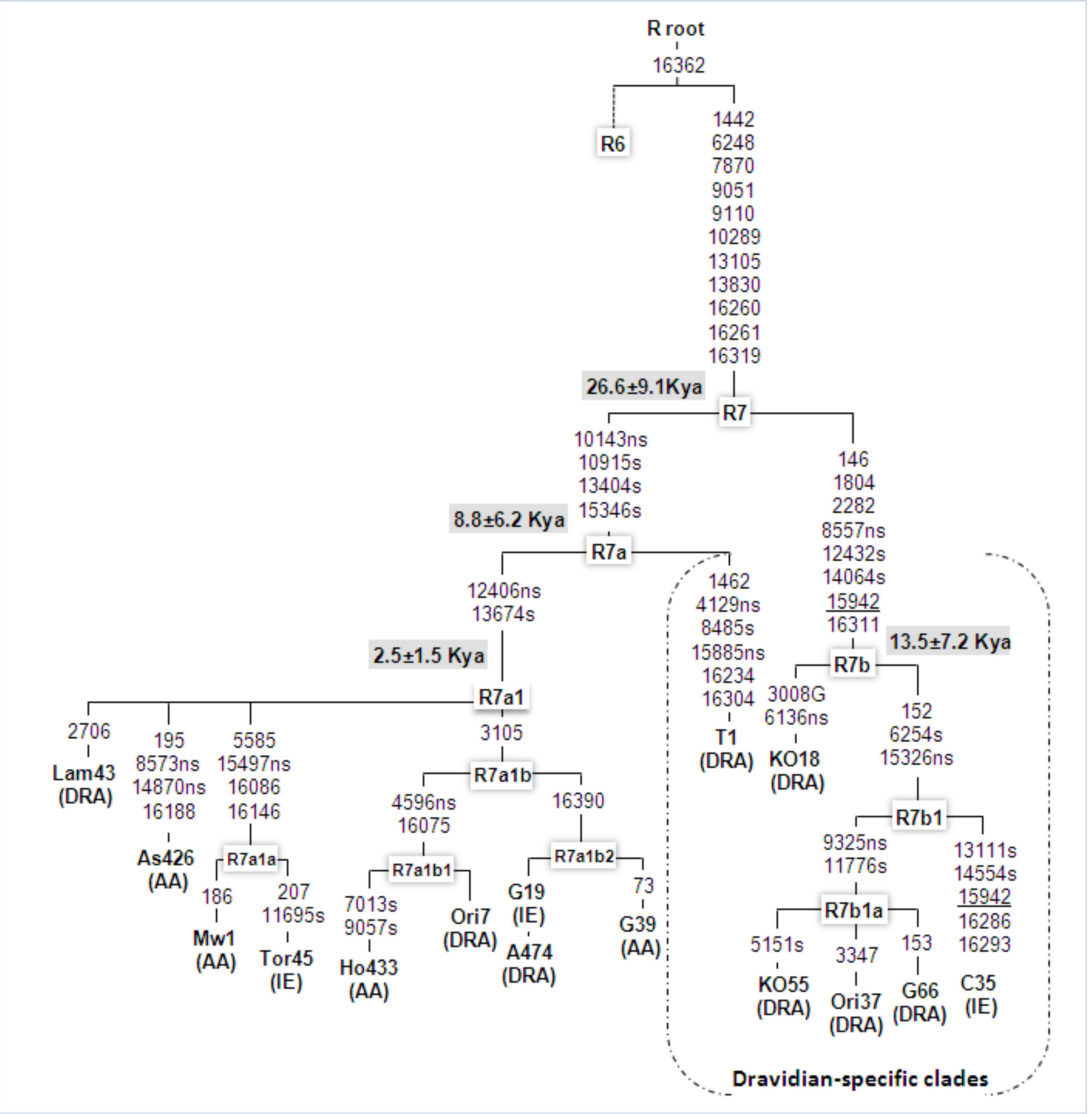
**The most parsimonious tree of haplogroup R7 complete mtDNA sequences observed in the Indian subcontinent.** This tree was redrawn manually from the output of median joining/reduced network obtained using NETWORK program (version 4.1) [34]http://www.fluxus-engineering.com. The samples were selected through a preliminary sequence analysis of the control region in order to include the widest possible range of R7 variation, language and geographical groups. Coalescent times were calculated by a calibration method described elsewhere [32]. 16182C, 16183C and 16519 polymorphisms were omitted. Suffixes A, C, G, and T indicate transversions, recurrent mutations are underlined. Synonymous (s) and non-synonymous (ns) mutations are distinguished. DRA-Dravidian, AA-Austro-Asiatic, IE-Indo-European. The ethnic affiliation of the samples is as follows: Lam, Lambadi; As, Asur; Mw, Mawasi; Tor45, Pakistan; Ho, Ho; Ori&A, Oraon; G19, Kanwar; G39, Santhal; G66, Gond; KO, Koya. Two sequences, T35 (Thogataveera) and C35 (Brahmin), were taken from the literature [4].

The inclusion of our 35 novel sequences (Table 1) into the phylogeny of haplogroup R allows the recognition of eight new subclades within six haplogroup R branches unique to the Indian subcontinent (Fig. 1, [see Additional file 1]). We refine here the internal topology of haplogroups R5, R6 and R8, and describe two novel sub-clades of hg R7, to be discussed below in detail. Subclade R5a is defined by a deletion at nucleotide positions (np) 522–523 and one control region mutation at np16266. R6a is defined by two control region substitutions (at sites 16129 and 16266). In haplogroup R7, two new subclades R7a and R7b can be identified (for details see further down). A new subclade of R8, called R8a, is defined by a single coding region substitution at np 5510. Haplogroup R30 splits into two subclades R30a and R30b, the former supported by ten coding region substitutions and the latter by 24 coding and control region mutations. Similarly, in haplogroup R31 a new subclade R31a can be distinguished by 17 control and coding region mutations. Coalescent estimates suggest an ancient branching pattern in hgs R30 and R31, dating back almost to the earliest diversification of the superhaplogroup R itself. This most probably occurred soon after the out of Africa dispersals into the Indian subcontinent [see Additional file 1].

**Table 1:** Geographical, Linguistic and Haplogroup Affiliations of Completely Sequenced mtDNAs.

Si No.	Sample code	Haplogroup	Population	Location	Lingustic affiliation
1	Kol77	R5a1	Koli	Gujarat	Indo-European
2	Ben46	R5a1a	Bengal	West Bengal	Indo-European
3	Up41	R5a1a	Middle caste	Uttar Pradesh	Indo-European
4	Kall43	R5a2b	Kallar	Tamil Nadu	Dravidian
5	K35	R5a2b	Kota	Tamil Nadu	Dravidian
6	Ori74	R5a2b2	Oraon	Orissa	Dravidian
7	Mo38	R5a2b3	Moor	Sri Lanka	Dravidian
8	Gu35	R5a2b3	Gujarat	Gujarat	Indo-European
9	Pn32	R5a2b4	Paniya	Kerala	Dravidian
10	Mal33	R5a2b4	Malayan	Kerala	Dravidian
11	Ko 5	R6a1a	Koya	Andhra Pradesh	Dravidian
12	Ko31	R6a1a	Koya	Andhra Pradesh	Dravidian
13	Lam43	R7a1	Lambadi	Andhra-Pradesh	Dravidian
14	As426	R7a1	Asur	Jharkhand	Austro-Asiatic
15	Mw1	R7a1a	Mawasi	Chhattisgarh	Austro-Asiatic
16	Tor45	R7a1a	Sindhi	Pakistan	Indo-European
17	Ho433	R7a1b1	Ho	Jharkhand	Austro-Asiatic
18	Ori7	R7a1b1	Oraon	Jharkhand	Dravidian
19	Ori37	R7b1a	Oraon	Orissa	Dravidian
20	A474	R7a1b2	Oraon	Jharkhand	Dravidian
21	G39	R7a1b2	Santhal	Bihar	Austro-Asiatic
22	G19	R7a1b2	Kanwar	Madhya-Pradesh	Indo-European
23	KO18	R7b	Koya	Andhra-Pradesh	Dravidian
24	KO55	R7b1a	Koya	Andhra-Pradesh	Dravidian
25	G66	R7b1a	Gond	Madhya-Pradesh	Dravidian
26	Ko74	R8a	Koya	Andhra Pradesh	Dravidian
27	Lam10	R8a1a1	Lambadi	Andhra Pradesh	Dravidian
28	Ko30	R8a1a2	Koya	Andhra Pradesh	Dravidian
29	Ko37	R8a1a2	Koya	Andhra Pradesh	Dravidian
30	CoB41	R8a1b	Konkanastha Brahmin	Maharashtra	Indo-European
31	CoB23	R30	Konkanastha Brahmin	Maharashtra	Indo-European
32	Sin49	R30a	Sinhalese	Sri Lanka	Indo-European
33	Pun47	R30b	Punjab	Punjab	Indo-European
34	Raj25	R31a1	Rajput	Rajasthan	Indo-European
35	Raj48	R31a1	Rajput	Rajasthan	Indo-European

**Figure 2:**
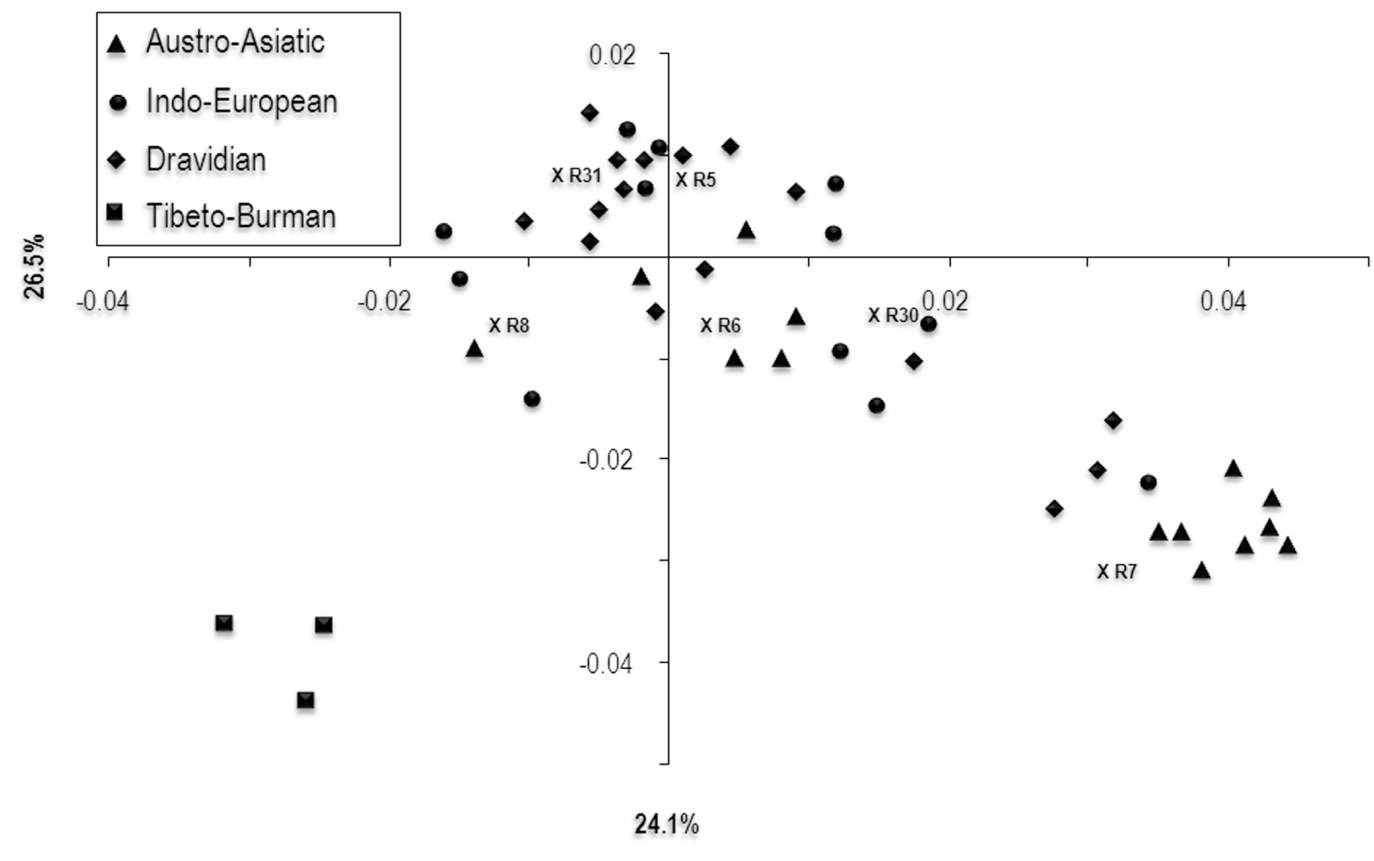
**Principal component (PC) analysis of R5-8, R30 and R31 lineages in Indian populations.** Munda group and a few Indo-European/Dravidian populations collected from Bihar, Jharkhand and Chhattisgarh states, predominantly cluster with haplogroup R7. Haplogroup frequencies were obtained from published sources [14] and our unpublished data.

Comparison of patterns of haplogroup distribution in relation to linguistic groups reveals that the frequency of the R7 clade is several times greater among AA (Munda) speakers than among Dravidian and Indo-European speaking populations (Table 2, [see Additional file 2]). Geographically, the distribution of R7 in India is centered on the AA "heartland" (Bihar, Jharkhand, and Chhattisgarh) [see Additional file 3]. Similar to R7, haplogroup R6 is significantly more frequent among the AA speakers than among other linguistic groups (Table 2, [see Additional file 2]). PC analysis based on frequency data of the hg R subclades confirms that the majority of Munda speaking populations cluster separately from others mainly because of higher hg R7 frequency (Fig. 2). However, only 50.6% of the variation can be explained by the first two principal components. Interestingly, hg R6 is placed within the main cluster, which is comprised of populations from all language groups. Based on these preliminary results we focused on R7 as a potential AA-associated marker.

**Table 2:** Frequency of Autochthonous R Subgroups Among Different Language Groups of India.

	R5	R6	R7	R8	R30	R31	Total Samples
**Austro-Asiatic**	1.12%	4.27%	5.90%	2.64%	0.61%	0.00%	983
**Indo-European**	3.62%	1.70%	0.58%	1.61%	2.63%	0.85%	2240
**Dravidian**	3.65%	1.69%	1.37%	1.64%	2.15%	0.32%	2190
**Tibeto-Burman**	1.74%	0.00%	0.00%	0.58%	0.58%	0.00%	172

In general, the elevated frequency of hg R7 among the AA speakers of India can be explained by two alternative scenarios. Firstly, one may consider a possible origin of R7 among AA (Munda) speakers, possibly already outside India. Under this scenario the presence of R7 in some Dravidian and Indo-European speaking communities would be explained by its later introgression from the Munda communities, or by language shift of some Munda speaking groups into Dravidian/Indo-European languages. Secondly, an origin of R7 may lie among non-AA populations of India, with the presently observable higher frequency of R7 among AA resulting from founder effect(s) due to random genetic drift. To test these two scenarios, we carried out a detailed analysis of R7 mtDNAs in populations speaking different subgroups of AA languages, as well as among IE and Dravidian-speaking populations of Indian subcontinent.

**Figure 3:**
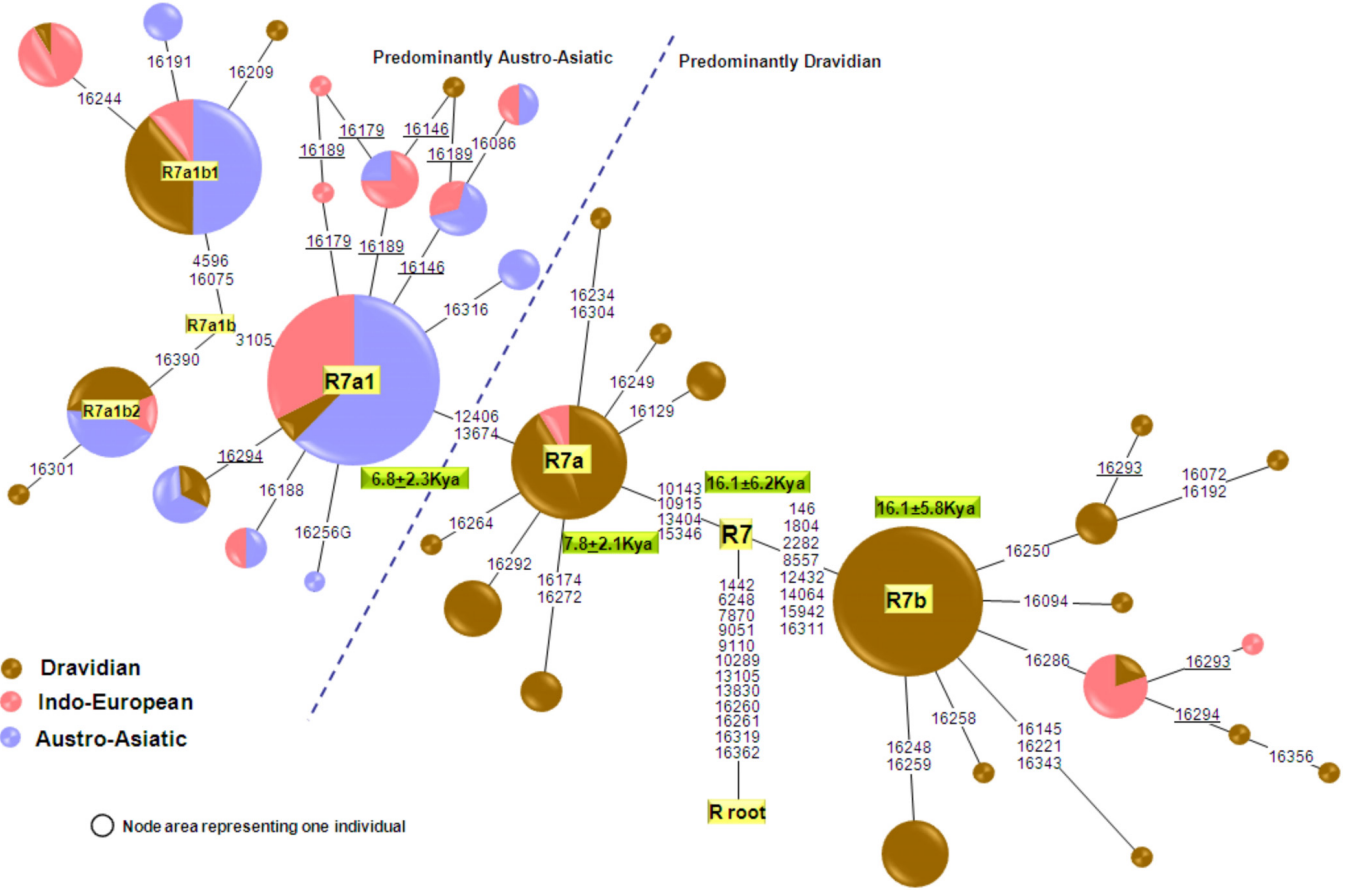
**The reduced-median network of 152 mtDNAs belonging to haplogroup R7.** Each sample represented on the diagram has been sequenced for the HVS-I region and genotyped for the coding region mutations that are indicated. Circle sizes are proportional to the number of mtDNAs with that haplotype. Recurrent mutations are underlined.

Complete mtDNA sequence-based topology of hg R7 divulges two deep-rooted subclades (Fig. 1). R7a is defined by four and R7b by six coding region mutations and, in addition, by two control-region substitutions (146 and 16311). We calculated the time to the most recent common ancestor (MRCA) for all R7 major sub-clades (Fig. 1 and 3, Table 3), applying different calibration methods [30,31]. All the AA individuals coalesce to the founder R7a1 that dates back to between approximately 3 Ka and 7 Ka ago, depending on the mutation rate used. The coalescent times of R7 variation among Dravidians and Indo-Europeans are older. In other words, the only R7 lineage found by us in AA speakers of India – R7a1 – is nested within the R7 lineages found among Dravidian and Indo-European speakers of India (Table 3).

**Table 3:** Coalescent times of hg R7 subclades estimated from HVS-I data.

Clade	Number of Samples	Motif (Coding region)	*rho *(ρ)	*sigma *(σ)	Time (SD)
**R7**	152	1442-6248-7870-9051-9110-10289-13105-13830	0.796	0.31	16.064 (6.260)
**R7(Austro-Asiatic)**	47	1442-6248-7870-9051-9110-10289-13105-13830	0.234	0.102	4.723 (2.059)
**R7(Indo-European)**	29	1442-6248-7870-9051-9110-10289-13105-13830	0.793	0.306	16.005 (6.185)
**R7(Dravidian)**	76	1442-6248-7870-9051-9110-10289-13105-13830	1.145	0.536	23.101 (10.822)
**R7a(Overall)**	107	10143-10915-13404-15346	0.389	0.102	7.848 (2.064)
**R7a(Dravidian)**	37	10143-10915-13404-15346	0.514	0.151	10.363 (3.037)
**R7a(Indo-European)**	24	10143-10915-13404-15346	0.5	0.24	10.090 (4.757)
**R7b(Overall)**	45	1804-2282-8557-12432-14064-15942	0.797	0.292	16.052 (5.891)
**R7b(Dravidian)**	39	1804-2282-8557-12432-14064-15942	0.744	0.268	15.006 (5.402)
**R7a1(Overall)**	86	12406-13674	0.337	0.115	6.805 (2.311)
**R7a1(Austro-Asiatic)**	47	12406-13674	0.234	0.102	4.723 (2.059)
**R7a1(Indo-European)**	23	12406-13674	0.522	0.246	10.529 (4.663)
**R7a1(Dravidian)**	16	12406-13674	0.375	0.153	7.568 (3.090)

**Figure 4:**
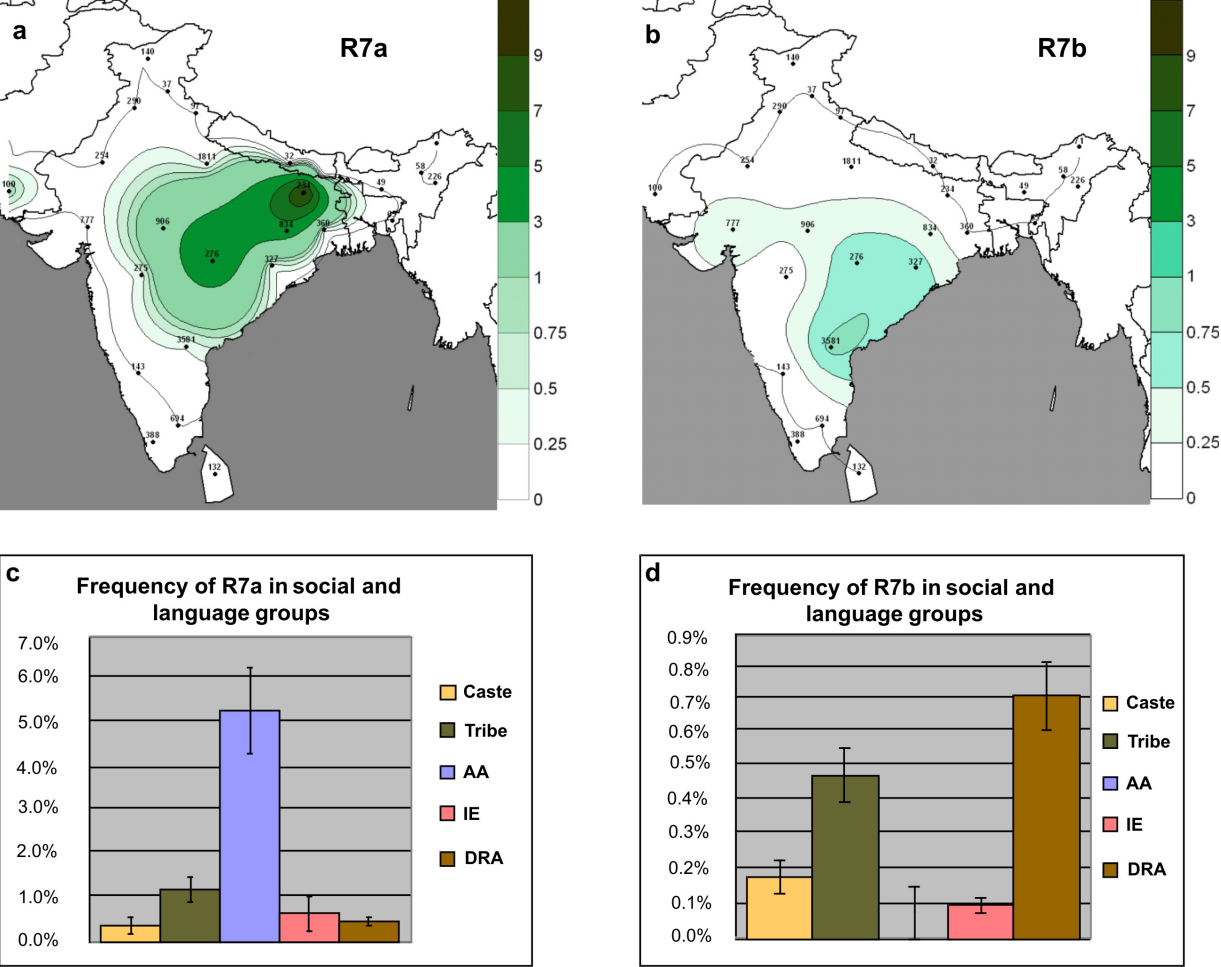
**The frequency distribution of R7a and R7b clades in Indian subcontinent.** The upper panel (a, b) shows the spatial distribution (%) of these clades in Indian populations. Isofrequency maps were generated by using Surfer7 of Golden Software (Golden Software Inc., Golden, Colorado), following the Kriging procedure. These isofrequency maps illustrate the geographic spread of the respective mtDNA haplogroups. It should be cautioned, however, that these illustrative maps should not be used to predict the frequency of the clade in geographical areas with missing data. The lower panel (c, d) depicts the frequencies of R7a and R7b in different social and language groups. DRA-Dravidian, AA-Austro-Asiatic, IE-Indo-European.

Geographically, the distribution of R7a frequency is concentrated towards Bihar, Jharkhand and Chhattisgarh States, while R7b has its frequency peak in Andhra-Pradesh (Fig. 4a and 4b). The frequency of R7a is higher among AA (Munda) speakers, while R7b is most common among Dravidian speakers from Andhra-Pradesh, although the overall frequency of R7b is much lower than that of R7a (Fig. 4c and 4d). A Mantel test showed a significant correlation between genes and geography for the Indian R sub-clades, but no such correlation for the relationship between genes and languages (Table 4). The spatial autocorrelation analysis favoured a clinal pattern for the distribution of hg R7 [see Additional file 4]. At the local (i.e. district) level, R7 is present in Bihar, Jharkhand, Chhattisgarh, Madhya-Pradesh and the northern districts of Andhra-Pradesh (Adilabad, Warangal and Khammam), whereas elsewhere in India it is virtually absent, including among other AA groups inhabiting Orissa and Maharashtra states [see Additional file 5].

**Table 4:** Mantel correlation test of Autochthonous R Subgroups to assess the significance of correlations between gene and geography, or language.

Haplogroup	Gene vs Geography	p	Gene vs Language	p
**R5**	0.1276	0.0475	0.1748	0.2
**R6**	0.2654	0.037	0.13248	0.19
**R7**	0.299	0.023	0.219	0.225
**R8**	0.211496	0.01753	0.23248	0.31
**R30**	0.189917	0.127	0.1348	0.28
**R31**	0.172	0.1873	0.141	0.25

The overall higher than average frequency of R7 among the AA speakers of India may superficially be seen as supporting the model that places the origin of this haplogroup among AA speakers, possibly even outside India, assuming the language phylum would have arisen elsewhere. Indirectly, such a scenario would be also supported by the Y chromosome evidence (haplogroup O2a, for details, see Introduction). However, the much higher diversity of R7a and R7b sub-clades among non Austro-Asiatic populations of India suggests that the source of haplogroup R7 is not among the maternal ancestors of all Austro-Asiatic tribal groups, but that they acquired this haplogroup via local admixture, together with the rest of the South Asian mtDNA lineages that make up their extant maternal lineage pool. Furthermore, the presence of only a single recent founder branch of R7, i.e. R7a1, among widely dispersed AA populations of India supports the founder event scenario by introgression of this lineage from the local non-AA populations before the range expansion of Munda speaking populations within India. If indeed R7 did have its origin among some so far unsampled populations of the present-day Myanmar or Cambodia, we would then expect to see different sub-divided AA populations losing by drift different sub-branches of R7a and R7b (to explain their reduced diversity), and the admixed Dravidian and Indo-European speaking populations would be expected to have obtained a subset of the R7 variation observed in AA speakers, which is not the case. While the occurrence of R7a1 among Dravidian and Indo-European-speaking populations living close to the AA populations (Fig. 3) could be explained by language shift or secondary admixture with AA speakers, sub-haplogroup R7b appears to be restricted to Dravidian-speakers of the southern part of India (Fig. 4b and 4d). Nevertheless, this haplogroup is also reported in two Indo-European populations (Kolcha and Rathwa) whose local tradition speaks about their ancient split from the Gond (Gondi subfamily of Dravidian language group) population of Central India and further migration to Gujarat. Thus, from the data and analyses shown here, it is most parsimonious to conjecture that R7 originated in India among non-AA, possibly in Dravidian speaking populations.

**Figure 5:**
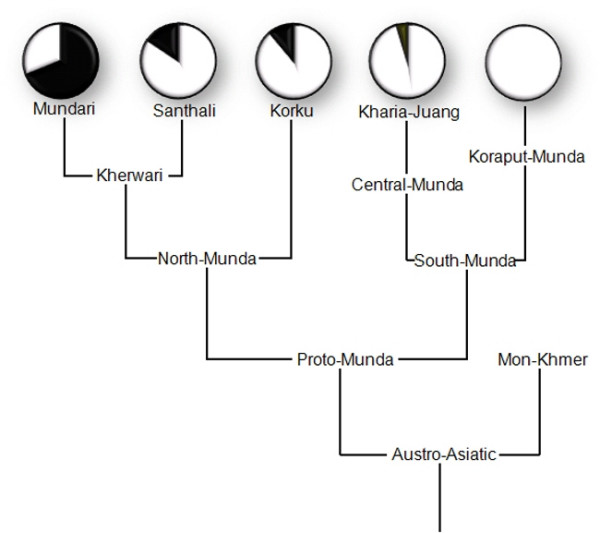
**The frequency distribution of haplogroup R7 in different branches of the Austro-Asiatic language family of India**[26].

To test further the two hypotheses, a Dravidian origin for R7 with admixture and founder effects, versus an external AA origin of R7, we examined whether the spread of R7 among the different Munda sub-groups in India, as defined by the language trees [26,27], is uniform. This would be expected if R7 was present among the ancestral AA speakers prior to the diversification of the language family into numerous branches. Consistent with the non-AA origin of R7, we found the distribution of R7a1 among AA populations to be profoundly skewed towards the Kherwari sub-branch of the North Munda languages which accounts for ~90% of the AA R7 samples (Fig. 5). Conversely, R7 is very rare in the South Munda group. It is completely absent in Koraput Munda speakers and marginally present only in the Kharia tribe of Madhya Pradesh (in total 3 out of 431 South Munda samples) (Fig. 5). This finding yet again strengthens the argument that only a subset of Indian AA groups has acquired one sublineage of R7a1 *in situ *after their arrival to Indian subcontinent from local non-AA groups through admixture. Thus, we fail to find from the evidence of the extant maternal lineage pool of the Austro Asiatic speakers of India any major lineages that show signs of potential origin outside India. Overall, the enigma of the origins and demographic past of the AA speakers in India remains, for while the East Asian contribution to their paternal gene pool seems evident, the maternal side of their genetic heritage appears to be autochthonous to Indian subcontinent. This suggests that introduction and spread of AA speakers into India involved a complex and sex-differentiated demography, involving both exogenous males and local females.

In brief, our high-resolution study of haplogroup R7 suggests that this haplogroup originated in India among non-AA population most probably Dravidian, and that the Munda (mainly Kherwari group) speaking populations have acquired a subset of it only relatively recently. The highest frequency of haplogroup R7 among Austro-Asiatic tribal groups can be explained, thus, by their regional admixture with other local Indian subcontinental populations followed by random genetic drift, rather than being a genetic marker of their own. The spread of R7 as well as other ancient sub-clades of haplogroup R in India follows predominantly the geographic rather than linguistic landscape of the subcontinent. The geographic correlations are further manifested in the distribution patterns of the sub-clades: R7a being more common in northern India while R7b is more frequent in the southern parts of the subcontinent. Because Dravidian speakers harbour all the twigs of R7 identified so far, the haplogroup may have arisen among the matrilineal ancestry of the present day Dravidian speakers. However, it is important to caution that autochthonous basal mtDNA lineages in South as well as Southeast and East Asia appear to be significantly more ancient than any linguistic reconstruction offers to present day language families. This would imply that linguistically significant relationships among Indian populations may be superimposed on, and masking, demographic events of much greater antiquity. Our results also remind us, once again, that phylogenetically established within-haplogroup diversity is more informative than mere frequency in establishing the direction of gene flow between populations, language groups and geographically defined regions.

## Methods

To refine the phylogeny of superhaplogroup R we sequenced complete mitochondrial genomes of 35 samples selected from different regions and language groups of India (Table 1). The results were incorporated into a phylogenetic tree [see Additional file 1]; for detailed tree for hg R7 see Fig. 1) together with previously published complete mtDNA sequence data from India [4]. For haplogroup R7 we performed a high-resolution survey of phylogenetically diagnostic markers, using information from complete mtDNA sequences. We studied ~12,000 samples collected from all over Indian subcontinent [see Additional file 6]. These samples cover all the language groups and most of the Indian states and union territories. The samples were screened for the presence of R7 mtDNAs based on HVS-I information (motif: 16260-16261-16319-16362). Previously this motif has, together with the restriction enzyme AluI cutting site polymorphism at np. 10143, been used to define haplogroup R20 [14]. However, with the support of new complete mtDNA sequences information the lineage with this HVSI motif was subsequently named R7 [4] and we follow this update of the nomenclature. Further, the identified R7 samples were analyzed for coding region markers by sequencing. Sequencing was carried out in ABI 3730 and 3730XL DNA Analyzers (Applied Biosystems, USA) and mutations were scored against the rCRS [33]. To minimize errors, both strands were double-sequenced. Principal component analysis (PCA) of R subgroups was performed using POPSTR, kindly provided by H. Harpending. Median-joining and reduced median networks were reconstructed with NETWORK program (version 4.1) [34]http://www.fluxus-engineering.com. Reduced median and median-joining procedures were applied sequentially. Coalescence time has been calculated between nucleotide positions 16090–16365 (HVS-I) considering one transition equals to 20,180 years [31], while for the coding region estimates we employed the rate calibrated by Kivisild et al. [32] considering substitution rate estimate for protein-coding synonymous changes of 3.5 × 10^-8^, which gives 6,764 years per synonymous transition. Standard deviation of the rho estimate (σ) was calculated as in Saillard et al. [35]. Haplogroup isofrequency maps were generated by using Surfer 7 of Golden Software (Golden Software Inc., Golden, Colorado), following the Kriging procedure. To determine whether language or geography has the strongest impact on genetic differentiation, spatial autocorrelation, SAAP [36] and Mantel [37] tests were performed using ARLEQUIN version 2.0 [38]. For Mantel test genetic distance matrixes were generated from ARLEQUIN, and geographic distance calculated from latitude and longitude information. For language groups linguistic distances (ranging from 10–100) assigned manually to each branch, based on published linguistic information and vocabulary match [26-28,39,40].

## Electronic database information

Accession numbers for data presented herein are as follows (for the complete mtDNA sequence accession numbers FJ004804-FJ004838 and for the HVS-I region sequence accession numbers FJ010662- FJ010785).

## Authors' contributions

GC, MK, EM, DS–R, VKS, AS, BPN, AK, NA, CBM, BT, SP, RR, PS, SB, SVe, SVa, IK, AB, DS, AS, MR, VC and AGR carried out the mtDNA genotyping. GC, MK, EM, DS–R, VKS, AS, and BPN carried out the mtDNA sequencing analysis. AT, KT and LS contributed to the analysis and interpretation of the data. AT provided complete sequence information of Sindhi sample. GC, MK, EM, MM and TK analyzed the data. TK, GC, MM and RV were responsible for conceiving and designing the study. GC, MK, MM, TK, RV, AT, RF, KT and LS wrote the paper. All authors read and approved the final manuscript.

## Supplementary Material

Additional file 1 Phylogenetic tree of 22 Indian complete mtDNA sequences of superhaplogroup R. The tree includes data reported [[4] and references there in] Suffixes A, C, G, and T indicate transversions, "d" signifies a deletion; recurrent mutations are underlined. 16182C, 16183C and 16519 polymorphisms are omitted in phylogenetic reconstruction. The sample code, geographic and linguistic affiliations are described in Table 1. The sub-tree of haplogroup R7 sequences is displayed in Fig. 2. Click here for file

Additional file 2 Haplogroup R5-8, R30 and R31 frequency plots with 95% credible regions. Data calculated from the posterior distribution of the proportion of a haplogroup/sub-haplogroup in the population. Linguistic affiliations of the populations are indicated by colors. Click here for file

Additional file 3 Map of Indian subcontinent depicting the spatial frequency distribution of mtDNA haplogroup R7. Isofrequency maps were generated by using Surfer7 Golden software (Golden Software Inc., Golden, Colorado), following the Kriging procedure. The spread of R7 in India is centered around the AA "heartland" (Bihar, Jharkhand, and Chhattisgarh). Dots indicate the sampling locations. Click here for file

Additional file 4 Spatial Autocorrelation Analyses Correlograms of haplogroup R7 in Indian subcontinent. The Moran's I coefficient was calculated with five distance classes in binary weight matrix. Significant values are shown as black (p < .05) whereas nonsignificant values as blank circles. Distances are given in Kilometers (KM's). Click here for file

Additional file 5 Map of India showing the frequency distribution (%) of haplogroup R7 at the district level. Only 2,200 samples were available at this resolution. Nevertheless, it is still evident that the frequency peak of R7 is observed in Bihar, Jharkhand, Chhattisgarh, Madhya-Pradesh and the northern districts of Andhra-Pradesh (Adilabad, Warangal and Khammam). Click here for file

Additional file 6 Details of the samples studied for hg R7 in the present study. Data shown are from the present work and from literature: [4,12,14,21,22,41-46]. Click here for file
