# Supplementary figures and images for: Phylogeography of mtDNA haplogroup R7 in the Indian peninsula
Source: BMC Evol Biol. 2008 Aug 4;8:227. doi: 10.1186/1471-2148-8-227 (PMC2529308; doi:10.1186/1471-2148-8-227)

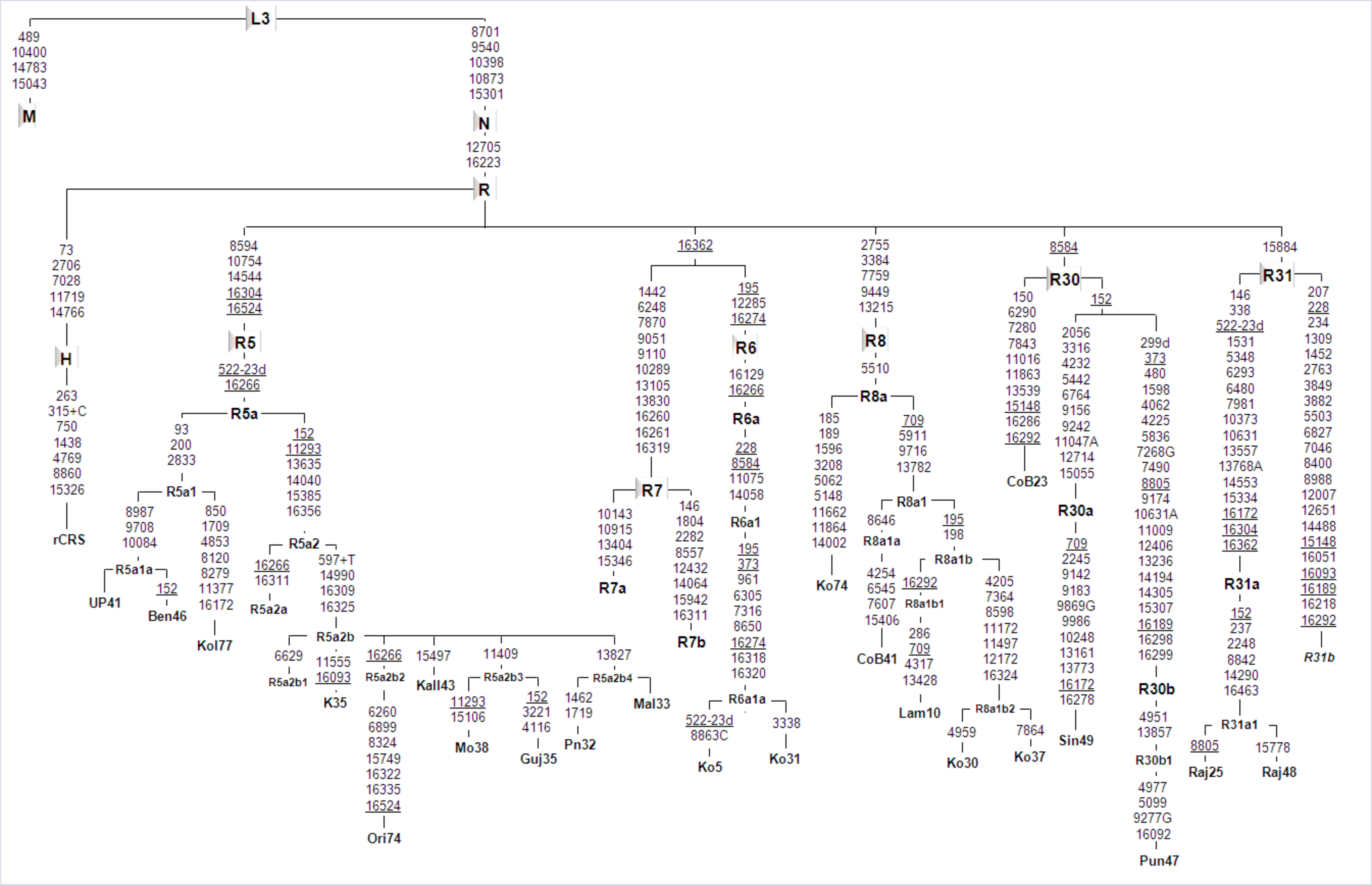

Supplement: Additional file 1 — Phylogenetic tree of 22 Indian complete mtDNA sequences of superhaplogroup R. The tree includes data reported [[4] and references there in] Suffixes A, C, G, and T indicate transversions, "d" signifies a deletion; recurrent mutations are underlined. 16182C, 16183C and 16519 polymorphisms are omitted in phylogenetic reconstruction. The sample code, geographic and linguistic affiliations are described in Table 1. The sub-tree of haplogroup R7 sequences is displayed in Fig. 2. [file 1471-2148-8-227-S1.jpeg]

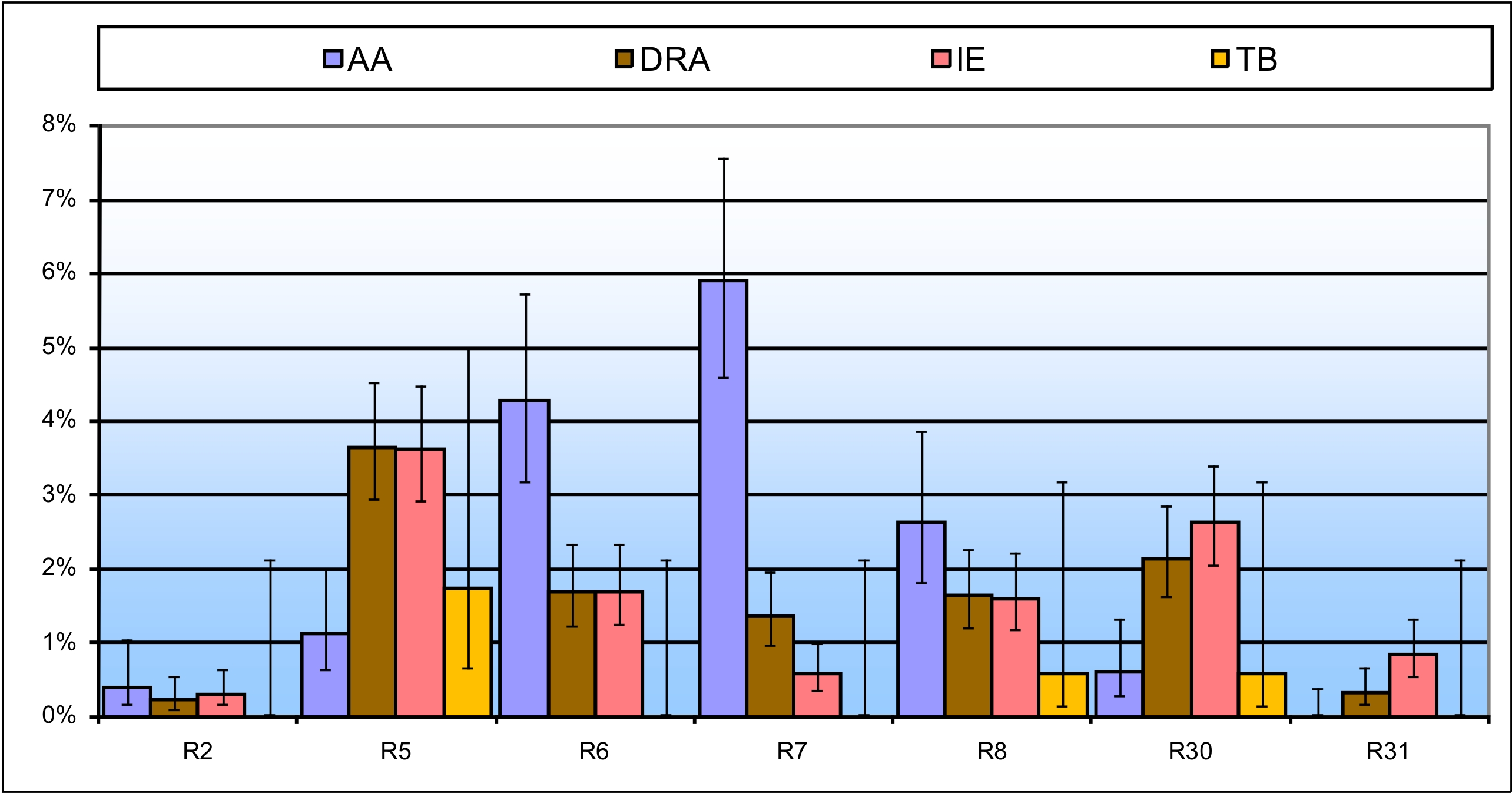

Supplement: Additional file 2 — Haplogroup R5-8, R30 and R31 frequency plots with 95% credible regions. Data calculated from the posterior distribution of the proportion of a haplogroup/sub-haplogroup in the population. Linguistic affiliations of the populations are indicated by colors. [file 1471-2148-8-227-S2.jpeg]

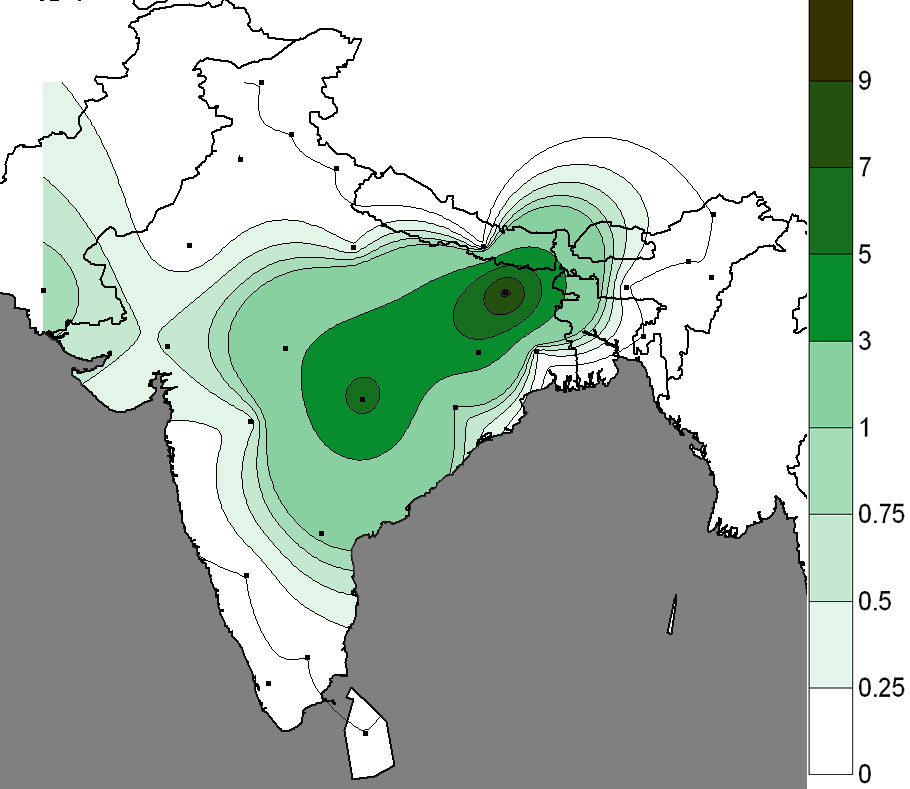

Supplement: Additional file 3 — Map of Indian subcontinent depicting the spatial frequency distribution of mtDNA haplogroup R7. Isofrequency maps were generated by using Surfer7 Golden software (Golden Software Inc., Golden, Colorado), following the Kriging procedure. The spread of R7 in India is centered around the AA "heartland" (Bihar, Jharkhand, and Chhattisgarh). Dots indicate the sampling locations. [file 1471-2148-8-227-S3.jpeg]

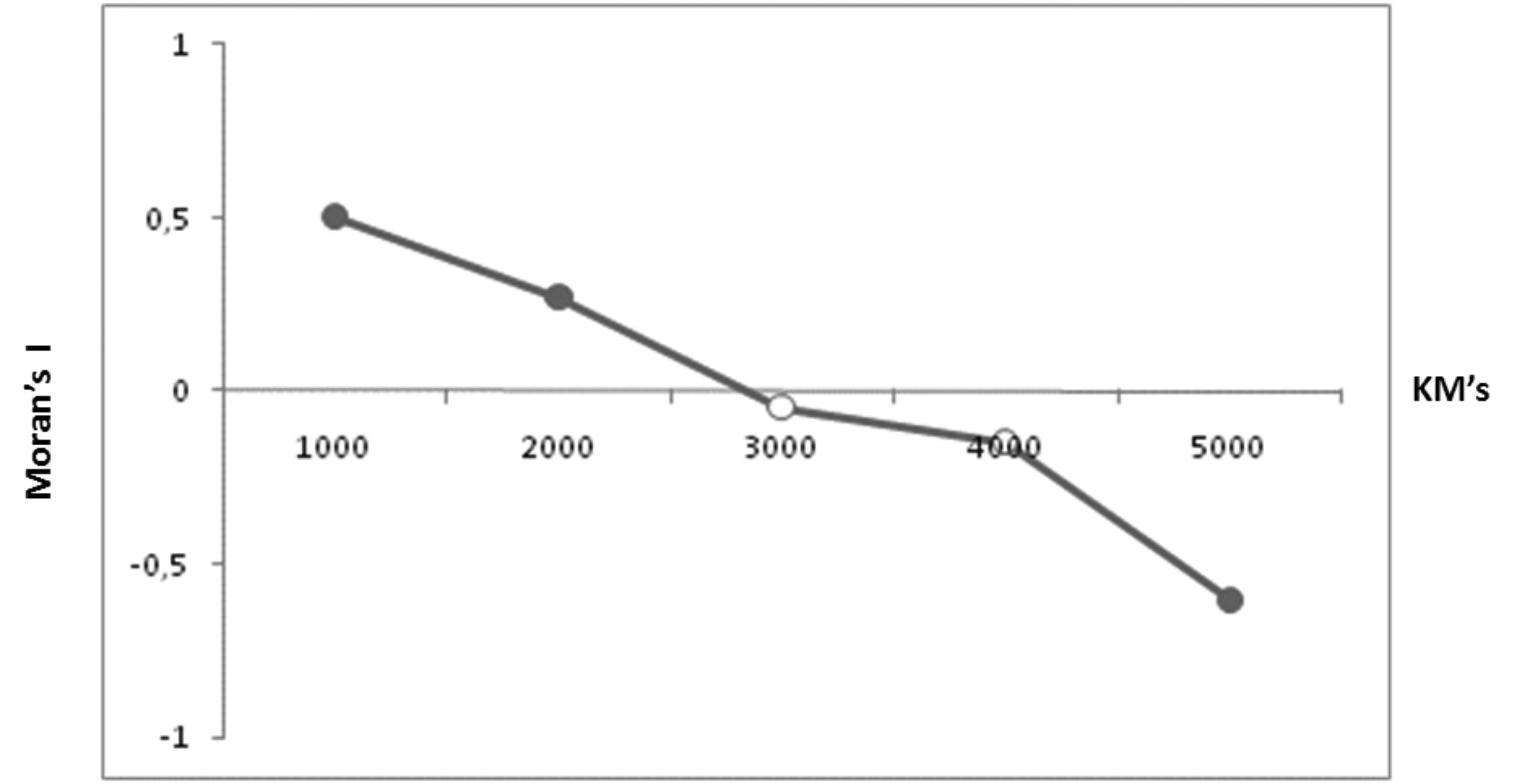

Supplement: Additional file 4 — Spatial Autocorrelation Analyses Correlograms of haplogroup R7 in Indian subcontinent. The Moran's I coefficient was calculated with five distance classes in binary weight matrix. Significant values are shown as black (p < .05) whereas nonsignificant values as blank circles. Distances are given in Kilometers (KM's). [file 1471-2148-8-227-S4.jpeg]

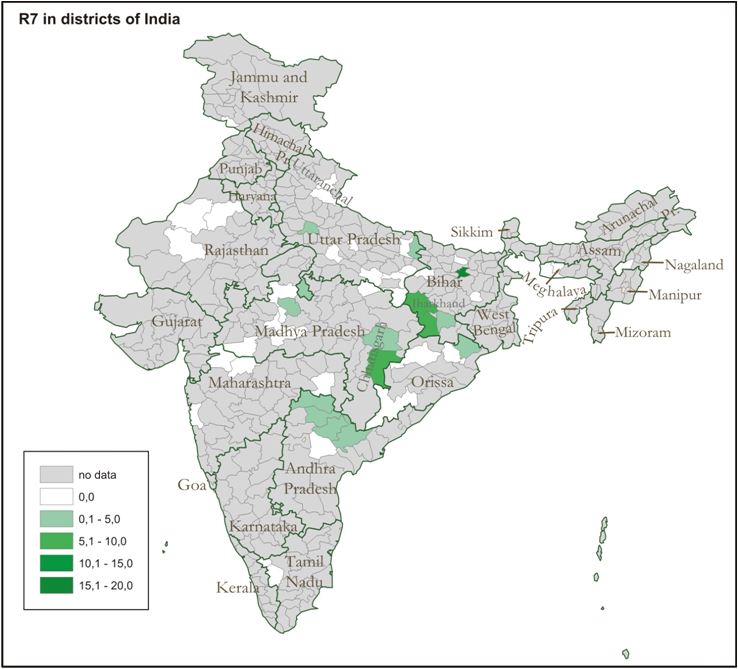

Supplement: Additional file 5 — Map of India showing the frequency distribution (%) of haplogroup R7 at the district level. Only 2,200 samples were available at this resolution. Nevertheless, it is still evident that the frequency peak of R7 is observed in Bihar, Jharkhand, Chhattisgarh, Madhya-Pradesh and the northern districts of Andhra-Pradesh (Adilabad, Warangal and Khammam). [file 1471-2148-8-227-S5.jpeg]
